# Tackling the possibility of extracting a brain digital fingerprint based on personal hobbies predilection

**DOI:** 10.3389/fnins.2025.1487175

**Published:** 2025-03-12

**Authors:** Cristina Andronache, Dan Curǎvale, Irina E. Nicolae, Ana A. Neacşu, Georgian Nicolae, Mihai Ivanovici

**Affiliations:** ^1^Sigma Laboratory, CAMPUS Institute, National University of Science and Technology Politehnica Bucharest, Bucharest, Romania; ^2^Faculty of Electrical Engineering and Computer Science, Electronics and Computers Department, Transilvania University, Brasov, Romania

**Keywords:** biometric authentication, brain-computer interface (BCI), category classification, electroencephalogram (EEG), emotion classification, event related potentials (ERP), hobby dataset, person identification

## Abstract

In an attempt to create a more familiar brain-machine interaction for biometric authentication applications, we investigated the efficiency of using the users' personal hobbies, interests, and memory collections. This approach creates a unique and pleasant experience that can be later utilized within an authentication protocol. This paper presents a new EEG dataset recorded while subjects watch images of popular hobbies, pictures with no point of interest and images with great personal significance. In addition, we propose several applications that can be tackled with our newly collected dataset. Namely, our study showcases 4 types of applications and we obtain state-of-the-art level results for all of them. The tackled tasks are: emotion classification, category classification, authorization process, and person identification. Our experiments show great potential for using EEG response to hobby visualization for people authentication. In our study, we show preliminary results for using predilection for personal hobbies, as measured by EEG, for identifying people. Also, we propose a novel authorization process paradigm using electroencephalograms. Code and dataset are available *here*.

## 1 Introduction

Electroencephalography (EEG) analysis has significantly advanced contemporary comprehension of the intrinsic mechanisms governing the human psyche (Cohen, 2017; Thompson, 2023; Brenninkmeijer, 2015). Regrettably, EEG data is characterized by inherent non-stationarity (Gramfort et al., 2013; Shen and Lin, 2019; Hine et al., 2017), presenting a significant challenge in the analysis and processing of this intricately variable signal. This challenge impedes the development of robust EEG applications (Saha and Baumert, 2020). However, recent research employing artificial intelligence (AI) (Hosseini et al., 2020; Wang et al., 2014; Gemein et al., 2020) lead to favorable outcomes in various applications. This suggests a potential direction for addressing the intricacies associated with detecting patterns in EEG data, that may otherwise elude human observation. Consequently, such AI-driven approaches hold promise in providing satisfactory results, irrespective of the paradigm employed in data collection.

Using EEG analysis in biometric applications represents a novel approach in the field of electroencephalogram classification, having only a few examples in the literature. In Wilaiprasitporn et al. (2020), the authors propose a new direction for person identification using EEGs. They use affective EEG classification, which is collected from subjects who passed through multiple mental states during acquisition. Namely, they train a combination of CNN and RNN on DEAP dataset (Chaudhary, 2023). In another work, Das et al. (2019) lay the foundation for EEG based identification by creating a state-of-the art neural network architecture based on CNN-LSTM combinations. They identify people in 2 scenarios: data collected with eyes closed and data collected while subjects kept their eyes open. Article Alyasseri et al. (2020) has a different approach, they use the flower pollination algorithm (FPA) and β-hill climbing (dubbed FPA β-hc by its authors) techniques to select the most relevant EEG channels for user identification. In another work, Thomas and Vinod (2018) prove the superior performance of power spectral density features of gamma band (30–50 Hz) in biometric authentication using EEGs. A challenge in the field is identifying individuals from acquisitions taken in different sessions and determining whether EEG permanence exists (Maiorana et al., 2015). In this regard, Maiorana (2020) explores the identification problem with a database recorded over a period of more than 1 year. Maiorana and Campisi (2017) take this type of analysis one step further by examining the effects of aging in EEG-based person identification. Using Hidden Markov Models, the authors demonstrate that they can successfully identify individuals in datasets recorded up to three years apart. Another common limitation in person identification is the dependence on the specific task performed during EEG acquisition. In order to overcome this challenge (Kumar et al., 2021) attempt to model biometric signatures independent of task/condition.

The main advantage of electroencephalogram approach in person identification lies in its unique combination of security and biometric specificity. EEG signals are highly individualized and extremely difficult to replicate or forge. This makes EEG an exceptionally secure method for identifying individuals (Bidgoly et al., 2020). Despite the promising premise, EEG analysis proves to be a strenuous task due to the signal's very low amplitude, difficult acquisition and non-stationary nature (Pinegger et al., 2016). However, with adequate acquisition quality, it provides several benefits. Firstly, it improves signal quality; which in turn enhances the ability to extract specific features which can be used in Brain Computer Interface (BCI) applications. Secondly, it presents detailed brain activity interpretation as it unfolds in real time.

Person authentication is highly correlated with person identification. This approach, in comparison to identification which assigns a unique identifier, considers people grouped by privilege access levels (e.g., using a badge in a corporation). Whereas identification focuses to answer the question “Who are you?,” authentication sets to answer “Are you who you pretend to be?” Thus, such applications can play a critical role in securing sensitive premises. In our work, we further develop this concept by incorporating results from both open-set and closed-set training scenarios.

New approaches in emotion classification tend to focus on the emerging field of neuromarketing (Duque-Hurtado et al., 2020). The fundamental aim of neuromarketing is to merge theories and methodologies from neuroscience with those from marketing and correlated fields like economics and psychology. This integration seeks to create neuroscientific valid interpretations of how marketing influences the behavior of target consumers (Lim, 2018). In Golnar-Nik et al. (2019), they study EEG spectral power potential in consumer preference prediction. The data was collected while participants watched mobile phones advertisements and they could choose to press a button meaning either like/dislike/buy or to press no button at all. Another interesting analysis was conducted by Aldayel et al. (2020). This study aims at bridging the gap between traditional market research, centered on explicit consumer feedback, with neuromarketing research, which focuses on implicit consumer responses. Nonetheless, classical emotion datasets are still used as benchmarks. Wan et al. (2023) develop an architecture, EEGformer, that can tackle several tasks including emotion classification, as tested on SEED dataset. As our work also focuses on preference degree classification, we hope that the results presented in this paper may be extended for future neuromarketing applications.

Given the current context of both machine learning and EEG analysis progress, recent work has focused on neural networks architectures tailored for BCI applications. Lawhern et al. (2018) propose an end-to-end neural network architecture. EEGNet is a compact CNN, which has the windowed preprocessed EEG time signal as input. The first layer is a 2D convolution layer where frequency filters are learned. It is followed by a 2D depth-wise convolution block with frequency-specific spatial filters. The third block consists of a separable convolution which mixes depth-wise convolution and point-wise convolution obtaining an optimal fusion between spatial and temporal features. Outputs of the third block are then fed to a dense layer which does the classification. Considering the compact architecture, end-to-end characteristic and good performance of the EEGNet, we considered it is fit for our classification purposes.

Thus, the neural network models proposed in this work were trained on EEGNet variations, with adjustments to filter sizes (to match sampling frequency), filter number (to obtain highest performance), and output layers (to fit class requirements).

At the same time, we make sure the user interest is in the center of the design. We set to detect an invariant digital brain signature, in the form of a response to a tailored stimulation, which is based on a mix of hobbies and reference categories. Also, the newly created dataset is publicly available. To validate the newly acquired dataset, we develop a fourfold experimentation paradigm. First, we aim to classify emotional responses corresponding to the following 3 labels: like, neutral, and dislike. Second, we set to classify the categories shown to each participant. The third and fourth direction are allocated to person authentication and identification respectively. For the former we propose a novel paradigm for security authorization. The above directions are implemented with convolutional neural network models, namely with variants of EEGNet (Lawhern et al., 2018). In summary, we use the newly created dataset on 4 different paradigms: emotion classification, macro-category classification (some similar categories were combined in order to increase training data), person authentication and person identification.

## 2 Experiment paradigm

### 2.1 Data acquisition

The experimental design was planned to maximize the brain response while maintaining subjects' engagement. In order to elicit a powerful EEG pattern, we used images of general hobbies, personal images (each participant was asked to bring a number of images with personal significance), as well as some reference categories. We considered that personal affinities and predilections tend to elicit more intense reactions and, thus, unique brain patterns. Further details regarding the selection of visual stimuli for our experiments can be found in Appendix I, and information about image authors is available in Appendix II.

Starting from current advancements in Event Related Potential (ERP) studies (Polich, 2007; Daliri et al., 2013), we developed an experimental paradigm that captures both visual (as measured at the occipital level) and cognitive activity. In order to obtain an intense cerebral activation, we chose stimuli to represent engaging images (hobbies, familiar landscapes or faces). These were intertwined with pictures without specific points of interest (stimuli with one single color, synthetic fractals, and repetitive patterns). The advantages of such an approach are the following: (i) the personalized experimental design is more likely to appeal to the participant and improve the chances of engaging in an eventual future similar application; (ii) the EEG response is expected to be emphasized due to the nature of the stimuli; (iii) the generality of the database categories opens the path for various EEG future applications.

**Figure 1:**
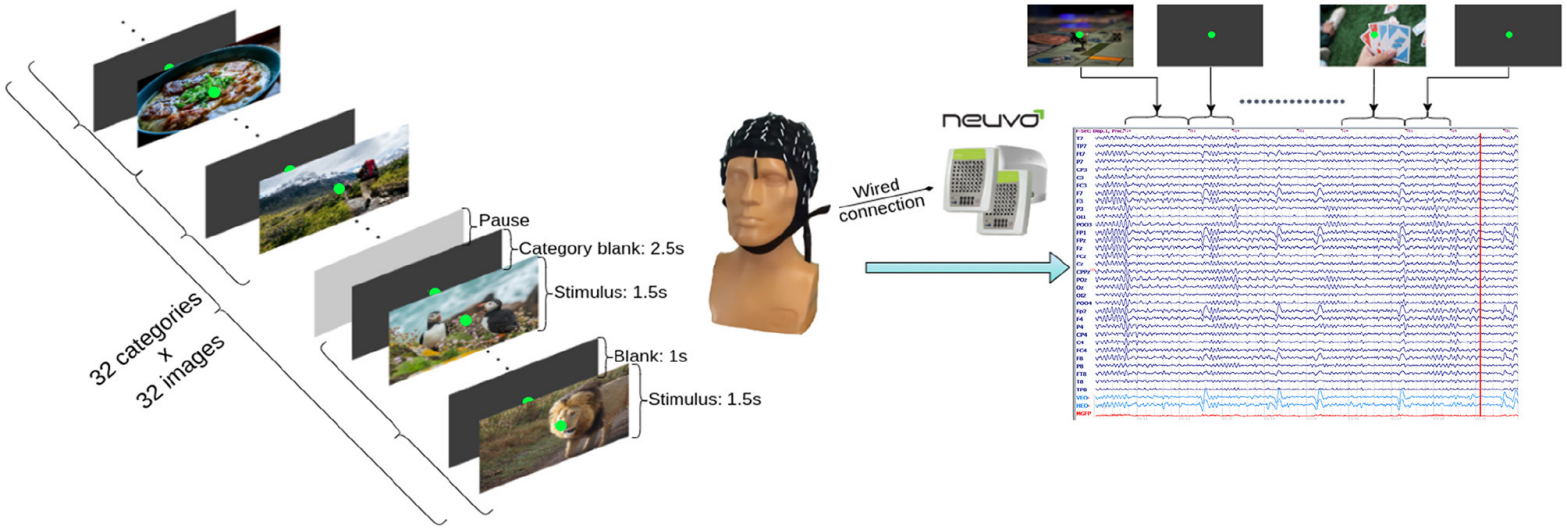
General diagram of the experimental design.

The acquisition of EEG data was performed under the guidelines of National University of Science and Technology Politehnica Bucharest ethical committee. Each participant was thoroughly informed of the nature of the experiment and how it will proceed. Also, all volunteers gave their written consent before participating in the study. The EEG experiment consisted in an ERP study with visual stimuli (Figure 1). The brain signals were recorded from 25 healthy participants (11 females and 14 males), in laboratory conditions. The age group was 21–42 years old, with a median of 24 years old. The data was acquired with 33 gel electrodes, in monopolar montage, with mastoid references. The EEG sensors were distributed according to the extended 10–20 system. The maximum acceptable impedance for the EEG sensors was 15 kΩ. In addition, eye movement activity was collected with 2 bipolar electrooculogram (EOG) electrodes corresponding to vertical and horizontal eye movement. The sampling frequency of the recording was 1 kHz. No hardware filters were used. More details on hardware and software can be found in Appendix III.

**Figure 2:**
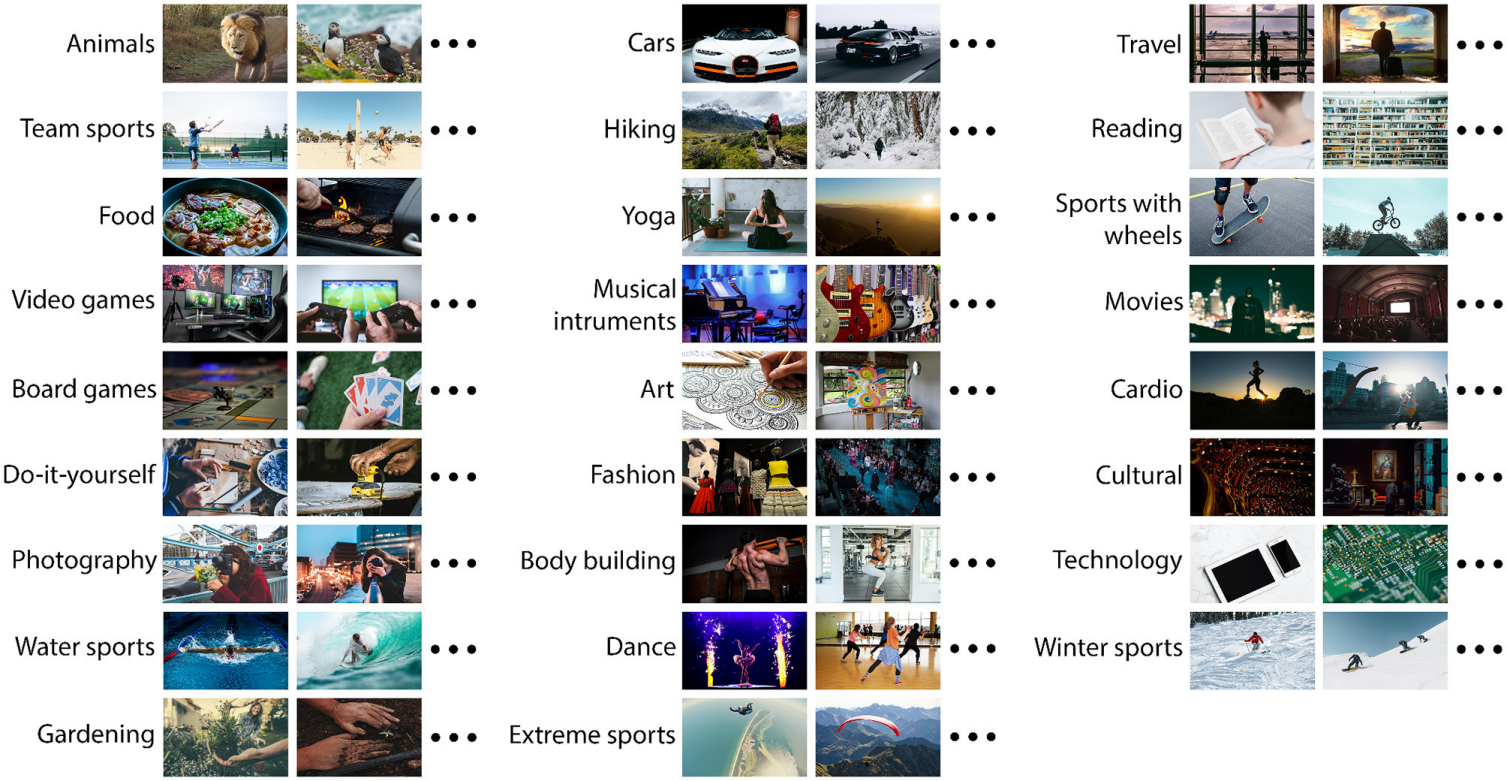
Hobby categories.

**Figure 3:**
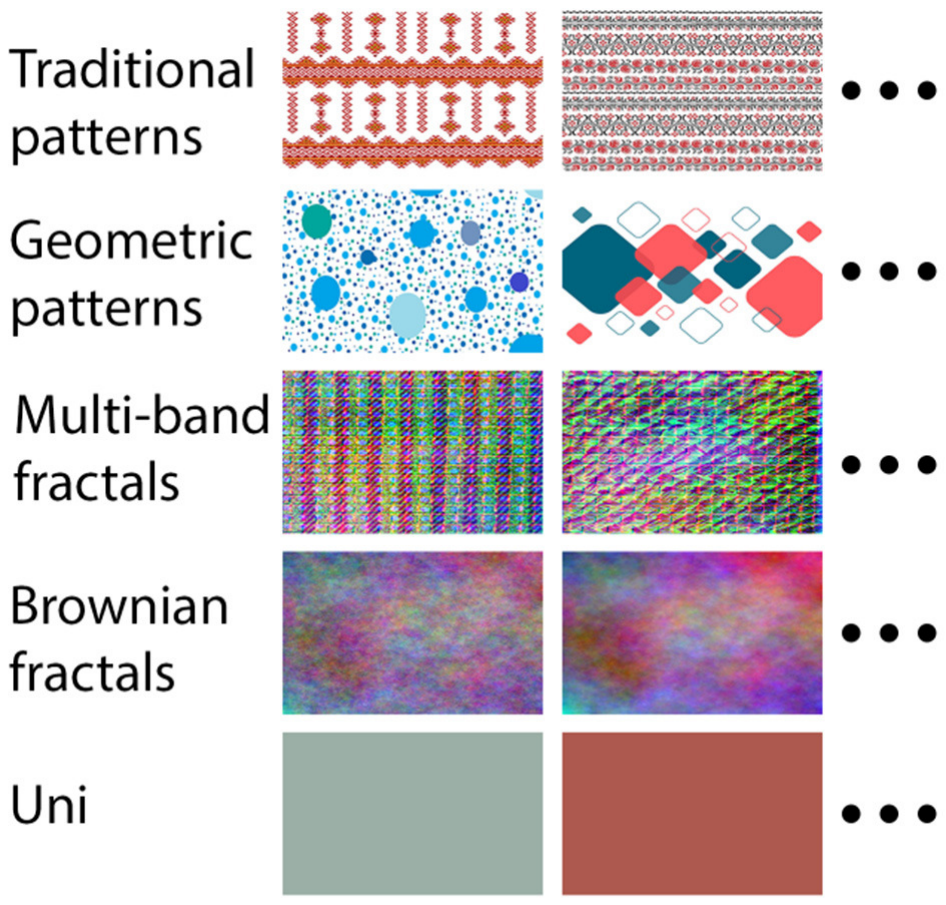
Reference categories.

Volunteers were requested to look straight and avoid additional eye movements during stimulus presentation. They were also asked to concentrate on the meaning of the picture shown—as to maximize the elicited reaction. The visual stimuli consisted in 32 image categories: 26 hobbies (Figure 2), 5 reference categories—images with no clear focus point (Figure 3), and one category containing personal images (brought by each participant). The personal category comprised pictures representing anything the volunteer found truly enjoyable e.g., family photos, images with friends, pets, art, etc. Those pictures were deleted as soon as the experiment was over in order to follow ethical guidelines regarding personal confidentiality. Each category comprised 32 images with 1,680 × 1,050 resolution, landscape oriented. All stimuli were presented in fullscreen mode and the subject sat at around 100 cm away from the screen. The images were carefully selected and mainly originated from free online platforms, such as *Unsplash*, *Freepik*, *Motivector* or *MBT Database*—details on image authors can be found in Appendix II. Stimuli categories only contained decent content and did not show any visible human faces (to avoid additional bias caused by preference or attraction). The only exception was the personal images category, which by nature is already biased and no constrain is needed. The experiment session was split equally in 32 blocks, with small breaks in between, each containing a hobby category. The 26 hobby categories were selected in concordance with a previous survey, which aimed to find out the most common hobbies and interests among people. A number of 96 respondents aged between 18 and 45 took part in the survey (see Supplementary material I).

Each one of the 1.024 images (32 categories × 32 images) are shown to the participant for 1.5 seconds. Pictures of the same category are shown one after another. To better differentiate the electrical brain response, we put a blank image lasting 1 second between pictures of the same category. Between categories the blank image is shown for 2.5 seconds (Figure 1). Blank images are used because they produce a standard brain response which is very attenuated compared to that of a non-blank image. The categories and the images in each category were always presented in the same order. Also, it should be noted that breaks were taken whenever the subjects wanted. The total duration of the whole experiment is averaged at 2 h, but the total visual stimulation lasted for: (1.5 s image visualization + 1 s resting state) × 32 images × 32 categories = 2,560 s = 42 min and 40 s. During acquisition, after each stimulus block, they were asked of their preference degree (like, dislike, or neutral), in response to the presented hobby. During this process, data was completely anonymized. After each category, participants were asked how much they liked it, as their hobbies. The distribution of these preference degree responses is presented in Table 1 for each category.

**Table 1:** Distribution of preference degree labels per category .

Animals:	1 – 9 – 15	Water sports:	0 – 9 – 16	Fashion:	8 – 10 – 7	Multi-band fractals:	18 – 6 – 1	Cultural activities:	4 – 8 – 13
Team sports:	7 – 10 – 8	Gardening:	9 – 10 – 6	Body building:	13 – 4 – 8	Brownian fractals:	17 – 6 – 2	Technology:	2 – 7 – 16
Food:	0 – 4 – 21	Cars:	13 – 8 – 4	Dance:	11 – 3 – 11	Uni:	17 – 8 – 0	Winter sports:	2 – 10 – 13
Video games:	10 – 5 – 10	Hiking:	4 – 2 – 19	Extreme sports:	5 – 9 – 11	Reading:	2 – 9 – 14		
Board games:	5 – 7 – 13	Yoga:	7 – 10 – 8	Romanian patterns:	16 – 6 – 3	Sports on wheels:	6 – 10 – 9		
DIY:	8 – 13 – 4	Musical instruments:	8 – 9 – 8	Colored patters:	12 – 11 – 2	Movies:	5 – 8 – 12		
Photography:	6 – 13 – 6	Art:	3 – 10 – 12	Travel:	2 – 4 – 19	Cardio:	6 – 9 – 10		

Each entry indicates the number of labels (dislike, neutral, like) a category received from all participants during the experiment. The table also shows the order the categories were presented: from top to bottom and from left to right. Category name: number of dislike labels – number of neutral labels – number of like labels

It can be noticed the categories “Food,” “Hiking,” and “Trips” were the most liked with over two thirds of participants giving them the label “like.” The most disliked categories are “Multi-band fractals,” “Brownian fractals,” and “Uni,” most likely due to their lack of meaning.

### 2.2 Data preprocessing

**Figure 4:**
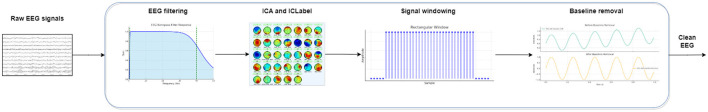
Pipeline of preprocessing steps.

In order to improve the quality of the raw signal, we designed a pipeline that removes noise and artifacts. These steps are precursory to data classification. The data processing pipeline depicted in Figure 4, consisted in the following steps.

#### 2.2.1 Signal filtering

The electrode-tissue interface introduces a significant DC offset (approx. 20–50 mV), which is 1,000 times higher than the usual EEG amplitude. Moreover, the signal tends to be altered by channel noise and high frequency artifacts. Consequently, a high-pass and a low-pass filter were applied to the newly collected EEG data. The high-pass filter is a FIR (finite impulse response) type filter. This filter has been set up with a cut-off frequency of 3 Hz, a transition band of [2.55, 3] Hz, and 0-phase shift to avoid any unwanted delays. The lowpass filter is an IIR (Infinite Impulse Response) Chebyshev Type II digital filter, which was used with a 49 Hz cut-off frequency. This setup helps to avoid the 50 Hz spike, which is caused by power line interference.

#### 2.2.2 Corrupted channel removal

Some channels are inherently noisier than others. This is caused by different electrode impedances, participant head shape, hair density and other factors. Thus, it is important to remove channels (here, we refer to entire channels) whose EEG signal is unrecoverable. In order to identify the corrupted sensors, we calculated the mean power of every channel and the median of those means. Outliers, with respect to the median, were to be removed from the data. After doing this type of verification, no channels needed to be eliminated from the dataset. This step was a preliminary one as the main noise removal was done with the help of Independent Component Analysis (ICA).

#### 2.2.3 Independent component analysis

The next processing step was artifact removal with ICA (Winkler et al., 2015). Artifacts in EEG data can come from the subject (e.g., eye movements, blinks, heartbeats, and muscle activity) as well as from the recording device (e.g., line noise, channel noise, etc.). To mathematically describe ICA algorithm, consider *M* signal vectors $\text{S}={\left({\text{S}}_{1},{\text{S}}_{2},\ldots ,{\text{S}}_{\text{M}}\right)}^{⊤}$, where each **S_i_** = (*s*_*i*1_, *s*_*i*2_, …, *s*_*iN*_) is a vector of *N* samples of the *i*-th signal, and each *s*_*ij*_ ∈ ℝ. The mixed signals can be represented by $\text{X}={\left({\text{X}}_{1},{\text{X}}_{2},\ldots ,{\text{X}}_{\text{M}}\right)}^{⊤}$, where each **X_i_** = (*x*_*i*1_, *x*_*i*2_, …, *x*_*iN*_). The mixing process for *M* signals involves a mixing matrix **A** ∈ ℝ^*M*×*M*^ with coefficients *a*_*ij*_ ∈ ℝ. The mixing process in matrix form is:


(1) $$\begin{array}{l} \text{X}=\text{A}\text{S} \end{array}$$

where **A** is the mixing matrix, **S** is the original signal matrix, and **X** is the matrix of mixed signals. The goal of ICA is to find the unmixing matrix **W** such that:


(2) $$\begin{array}{l} \text{W}={\text{A}}^{-1} \end{array}$$

The demixing process is:


(3) $$\begin{array}{l} \text{Y}=\text{W}\text{X} \end{array}$$

where $\text{Y}={\left({\text{Y}}_{1},{\text{Y}}_{2},\ldots ,{\text{Y}}_{\text{M}}\right)}^{⊤}$ is the matrix of estimated independent components.

**Figure 5:**
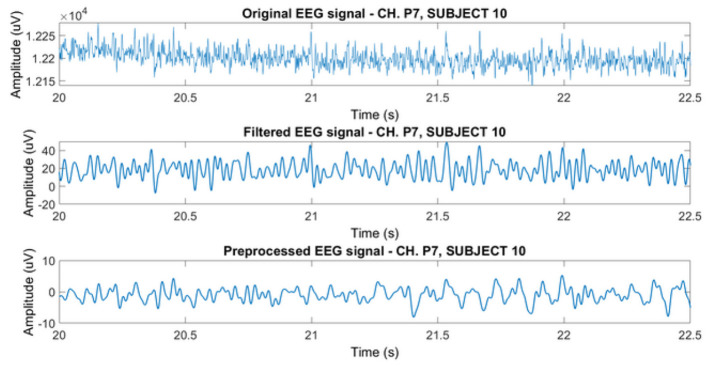
The impact of EEG signal preprocessing pipeline. From top to bottom: the raw signal; the signal after 3 Hz high pass and 49 Hz low pass filtering; and the signal after ICA filtering. The signal is extracted from the “animals” category (subject 10 and channel P7).

**Figure 6:**
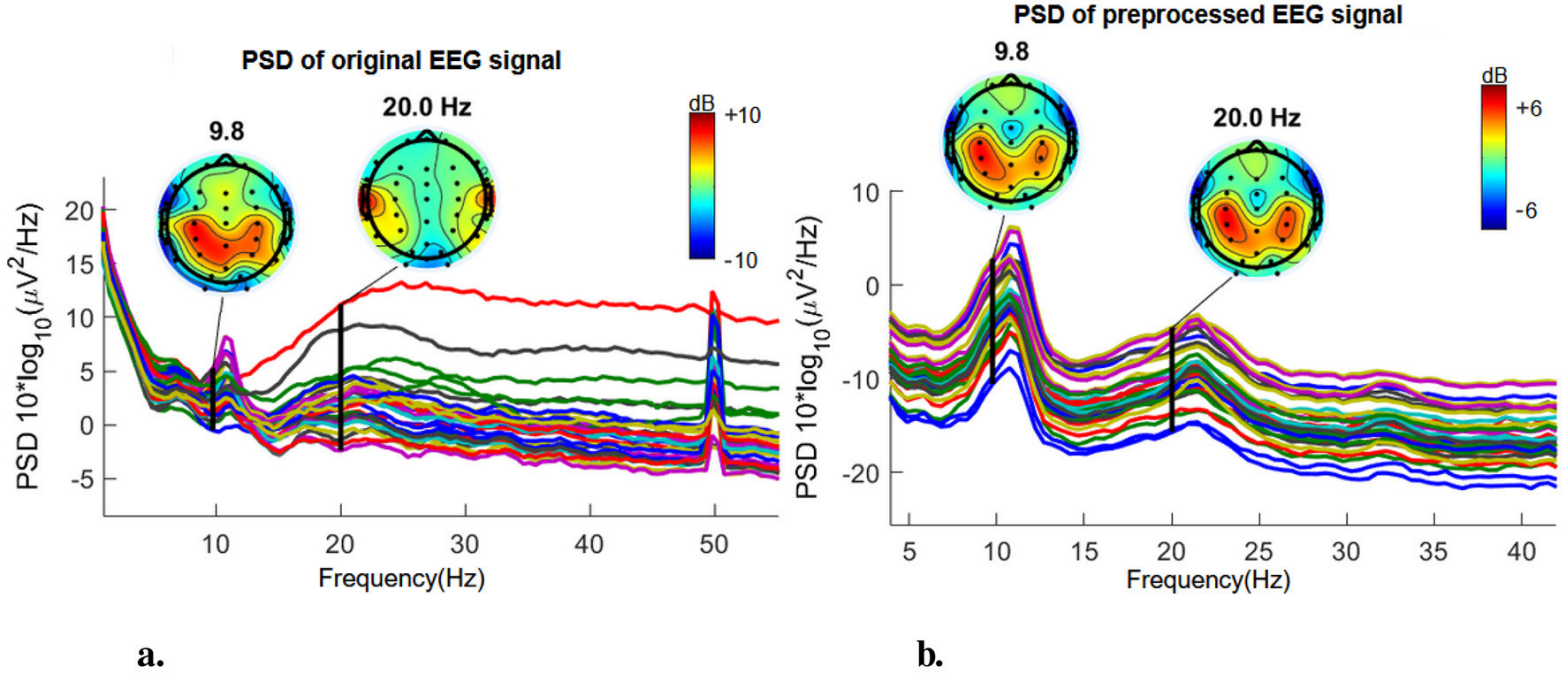
Power spectral density (PSD). Subject 10, category: animals. **(A)** Original. **(B)** Preprocessed.

Each estimated component vector **Y_i_** is given by:


(4) $$\begin{array}{l} {\text{Y}}_{i}={\text{W}}_{i}^{⊤}\text{X} \end{array}$$

where **W**_*i*_ is the *i*-th row of the unmixing matrix **W**, and **Y**_*i*_ = (*y*_*i*1_, *y*_*i*2_, …, *y*_*iN*_) represents the *i*-th demixed signal vector. In our use case, we chose *M* = 33 as the maximum possible number of components, i.e., the number of channels used for acquisition. After applying ICA, we classified the resulting 33 components as follows: *brain, muscle, eye, heart, line noise, channel noise*, and *other* using ICLabel (Pion-Tonachini et al., 2019), an automated electroencephalographic independent component classifier. ICLabel has undergone training through an Artificial Neural Network (ANN) on spatio-temporal characteristics of more than 200,000 independent components (ICs) derived from over 6,000 EEG recordings. This process also included the annotation of matching component labels for more than 6,000 of these ICs. The non-brain components were then subtracted from each EEG channel using a weight matrix (as each component has varying contribution on the overall signal). For example, electrodes located on the frontal lobe are prone to artifacts from eye blinks, thus *eye* components weigh more in the signals from frontal electrodes than in those coming from the central lobe. The signal's noise and artifact caused variation is diminished after filtering and preprocessing. The signal jitter is reduced, as exemplified in Figure 5, and the PSD slope acquires its 1/*f* shape with dB variations no higher than 15 Hz (Figure 6).

#### 2.2.4 Data epoching

After ICA, the next step was segmenting the EEG data corresponding to the visualized image. Also, during this phase we applied baseline correction for each epoch, where the baseline represents the 500 ms of blank image shown before each stimuli. After epoching, we refined the dataset further by using two criteria: peak to peak amplitude and variance (details in the following subsection).

#### 2.2.5 Epoch removal

Despite extended data processing, some EEG segments remain irretrievable. Also, ICA and IClabel have their limitations and we decided to double check the quality of the epochs. Thus, the epochs, which were obtained in the previous step, were verified and removed (if necessary) by a min-max and a variance criterion. More precisely, we removed epochs which had peak-to-peak amplitude spikes bigger than 150 μ V and a variance bigger than the average of all epochs. The later was done by computing the variance of each epoch in every acquisition. For every acquisition, we selected a threshold defined as the sum between the variance considering the 90^*th*^ percentile and 3 times the difference between the 90^*th*^ and 10^*th*^ percentiles. Epochs falling out of this range (i.e., have variance bigger than the defined threshold) were eliminated. Thus, 16 out of the 24 subjects needed to have some epochs removed. In general, we eliminated between 1 to 2 epochs for about 2 image categories per subject. After these preprocessing steps, we remain with 24 subjects out of the initial 25. The reason was that participant 25's recordings were significantly noisier than the others.

## 3 Experimental scenarios and results

Depending on each particular task, we used a slightly modified version of the EEGNet neural network architecture. Tuned hyper-parameters include output layer dimension, batch size, normalization rate, dropout and dropout type. In addition, the dimension of the first convolutional layer has been set according to our sampling rate of 1 kHz (length changed to 256). All presented results correspond to the mean performance over a 5 fold cross validation. The proportion between train and test has been 80%–20%.

**Figure 7:**
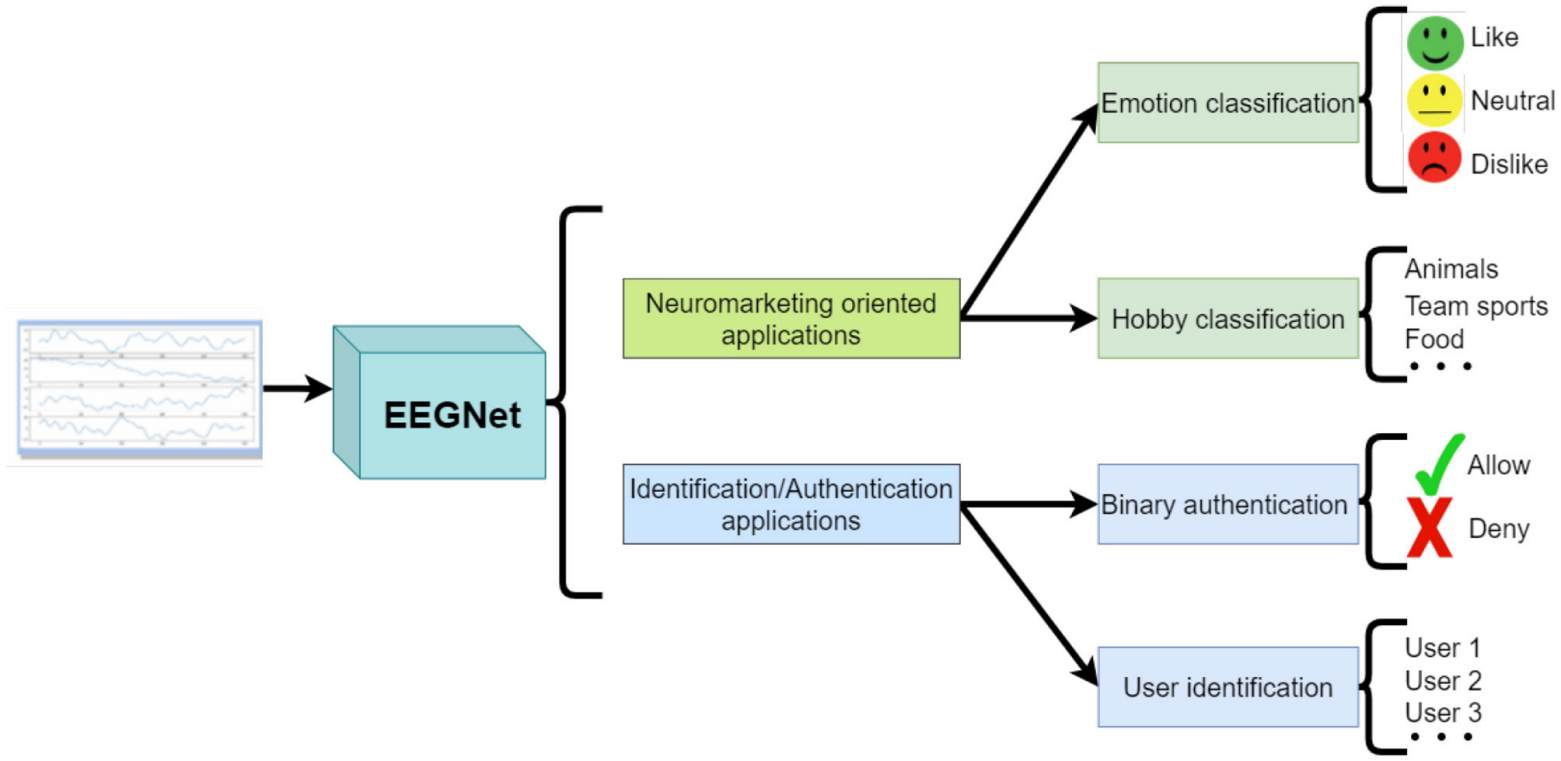
Experimental scenarios diagram.

The paradigm for the 4 employed scenarios is depicted in Figure 7.

### 3.1 Emotion classification

The first task consisted in classifying the preference degree of each user in response to the 32 categories. In this case, their respective labels are: *like, dislike*, and *neutral*. As images depict the same subject (hobby, reference category, or personal category), we premised that each image in a certain category has the same label as the entire category. Thus, for each subject there are up to 1,024 labeled signals (some subjects have less due to epoch removal in the preprocessing stage). Considering that labels are not homogeneous, as seen in Table 1, we used a balanced accuracy metric to measure the performance.

For the emotion classification task, 3 methods were employed. SVM, pyRiemann (Congedo et al., 2017), and EEGNet results are presented in Table 2. The second method, pyRiemann is an EEG classification approach based on Riemannian geometry. It implies projecting data onto a manifold space and calculating Riemannian distances between points in order to assign their class by proximity. It can be noted that EEGNet vastly outperforms the other 2 methods. Also, EEGNet and in some regard pyRiemann (Congedo et al., 2017) offer relative consistent results across the 24 subjects. In comparison, when applying SVM, there are users whose EEG data cannot be classified above random level (e.g., U1, U12, U18, etc.). Tables with additional results are offered in supplementary material (Appendix IV). These include extended performances on each class for the 3 methods and results obtained when training a model for each user.

**Table 2:** Mean emotion classification accuracy between the 5 folds.

	**SVM**	**pyRiemann**	**EEGNet**
U1	33.79 ± 2.45	47.76 ± 2.27	70.61 ± 3.66
U2	44.22 ± 4.23	56.47 ± 4.30	80.88 ± 2.32
U3	51.52 ± 2.71	60.36 ± 7.52	81.36 ± 2.34
U4	44.05 ± 3.30	54.34 ± 1.64	76.50 ± 3.86
U5	44.82 ± 3.51	54.88 ± 3.41	83.01 ± 2.78
U6	42.67 ± 3.44	51.37 ± 2.60	87.27 ± 3.61
U7	46.33 ± 2.08	57.00 ± 2.67	89.33 ± 2.10
U8	45.07 ± 1.85	62.76 ± 4.45	87.38 ± 1.27
U9	36.33 ± 1.95	46.92 ± 2.70	64.44 ± 6.97
U10	37.60 ± 1.95	55.86 ± 4.14	91.40 ± 3.64
U11	44.92 ± 2.89	50.88 ± 2.63	85.46 ± 3.84
U12	32.65 ± 4.76	47.51 ± 3.36	83.48 ± 2.52
U13	54.39 ± 1.92	57.11 ± 6.04	92.33 ± 3.15
U14	46.67 ± 4.54	65.31 ± 4.28	89.37 ± 2.88
U15	41.47 ± 1.82	51.08 ± 2.55	81.86 ± 3.21
U16	30.24 ± 3.20	38.81 ± 1.78	70.47 ± 2.65
U17	45.51 ± 3.19	45.90 ± 2.72	83.89 ± 5.03
U18	32.75 ± 3.19	55.81 ± 3.51	84.46 ± 3.19
U19	37.89 ± 1.99	42.87 ± 3.08	80.58 ± 6.89
U20	39.69 ± 1.45	48.85 ± 2.27	67.92 ± 1.79
U21	42.99 ± 3.51	55.34 ± 6.04	86.29 ± 2.50
U22	39.71 ± 3.41	43.36 ± 6.16	91.93 ± 3.12
U23	46.10 ± 3.91	47.36 ± 3.86	80.47 ± 4.64
U24	40.72 ± 1.91	52.35 ± 3.42	87.41 ± 3.68
Overall	41.74 ± 0.25	52.07 ± 0.68	82.44 ± 1.27

The experiment was done with 3 classes: *dislike, neutral*, and *like*. Results are presented for each user separately. Three classifiers were tested: SVM, pyRiemann (Congedo et al., 2017) and EEGNet (Lawhern et al., 2018).

### 3.2 Macro-category classification

This task proved to be especially difficult as it implied generalizing between a significant number of classes (i.e., 32) as well as a significant number of different persons (i.e., 24). EEG data is notoriously difficult to classify even if it is recorded from the same subject and during the same type of task. Nonetheless, we tried to classify the 32 categories with both SVM and EEGNet but results were less than satisfactory, barely surpassing chance level . Because data was not sufficient for such a complex task, we increased the number of training examples by aggregating some categories into macro-categories. For example, we considered *water sports, hiking*, and *body-building* as part of an overarching aggregate category called physical activity. By employing this approach we increased the level of abstraction, which in turn encourages the model to generalize across both subjects and ideas. The proportion of 80% train and 20% test was kept across both individual labels and subjects. Thus, all subjects had samples in training and testing. All macro-categories have uniform representation in train data. Results are promising, as we reached 83.77% accuracy with relative low deviation between folds, see Table 3. In Supplementary material IV, the aggregate category type of classification is also reported for 3 and 5 macro-labels.

**Table 3:** Label classification (macro-category classification).

**Aggregate category**	**Composing categories**	**Accuracy**	**Overall performance**
Physical movement	Water sports Hiking Body building	79.34 ± 4.21%	83.77 ± 1.50%
Reference	Fractals Brownian fractals Mono-color	86.38 ± 1.75%	
Serenity/calm	Animals Personal images Musical instruments	85.25 ± 2.05%	
Games	Video games Team sports Board games	84.26 ± 2.39%	

### 3.3 Binary authentication (authorization process)

For this task, we considered the following scenario. Imagine that there are special premises where only a certain group of people should be allowed entry. We name this group the “allow” group. Any other person should have the entry request refused. We name this complementary group the “deny” group. Thus, each person will go through an authorization process that outputs a binary response: either “allow” or “deny.” This approach can be implemented in two variants. One supposes that all subjects are known and, therefore, samples from all subjects are fed to the neural network. We will call this authentication paradigm “closed set.” For this task we considered part of users in the “allow” group and the rest in the “deny” group (as shown in Table 4). In order to validate the performance, we experimented with 3 group partitions. The first is an equal distribution between the 2 classes, second more users in the “deny” group and lastly more users in the “allow” group. In concordance with the previous tasks, we reported results from 5-fold cross validation testing. All reported metrics: accuracy, precision, recall, and F1 score offer good results. It is worth mentioning that a balanced training set, as it is presented in the first case of the closed set scenario, gives the best results with respect to all considered metrics.

**Table 4:** EEG based authentication performance [%] (2 classes representing “Allow” or “Deny”); “Closed Set”— training and testing is done with EEG epochs from all users; “Open Set”—testing on users whose EEG data was not present during training, thus, they can only be part of “Deny” category.

	**Closed set**		**Open set**		
	**Allow**	**Deny**	**Validation**		**Test**
			**Allow**	**Deny**	**Deny (exclusive)**
	**12 Users**	**12 Users**	**11 Users**	**11 Users**	**2 Users**
Accuracy:	99.76 ± 2.90e-3		87.02 ± 6.55e-2		93.31 ± 5.67e-2
Precision:	99.73 ± 2.80e-3		88.94 ± 3.69e-2		–
Recall:	99.67 ± 2.90e-3		87.02 ± 6.54e-2		–
F1 score:	99.76 ± 0.20e-2		86.67 ± 7.17e-2		–
	**6 Users**	**18 Users**	**9 Users**	**9 Users**	**6 Users**
Accuracy:	89.12 ± 4.29e-2		87.91 ± 4.49e-2		87.25 ± 0.13
Precision:	92.25 ± 1.53e-2		88.82 ± 3.92e-2		–
Recall:	79.36 ± 9.30e-3		87.90 ± 4.49e-2		–
F1 score:	82.17 ± 9.23e-2		87.81 ± 4.58e-2		–
	**18 Users**	**6 Users**	**10 Users**	**10 Users**	**4 Users**
Accuracy:	81.67 ± 2.89e-2		86.17 ± 4.10e-2		88.44 ± 9.22e-2
Precision:	77.12 ± 1.90e-2		87.05 ± 3.44e-2		–
Recall:	82.06 ± 1.77e-2		86.17 ± 4.10e-2		–
F1 score:	78.08 ± 2.23e-2		86.06 ± 4.22e-2		–

The other scenario variant assumes that the EEG from people in the “allow” group should be recognized even if the model is tested with EEG from new subjects (i.e., the neural network did not get the chance to train on them). We name this authentication variant “open set.” This way we emulate an open world environment where impostors are likely to appear. Therefore, the impostors will present an EEG signature that never appeared during the training process. Thus, in order to validate the model, we used a couple of users exclusively for testing. Ideally, the test users should always be labeled as “deny.” As there is no false “deny” or true “allow;” precision, recall and F1 score are not reported for the test set. In order to assess the model's capacity to perform an authentication task, we explored 3 ways of splitting the data in “allow,” “deny,” and “deny” for test only. To ensure consistency between the train and validation datasets, we split in an 80%–20% ratio for each subject (except for the ones kept exclusively for testing). This approach guarantees that no “allow” EEG signals are exclusively present in the test data. The configurations and results are presented in Table 4. Unlike the “closed set,” this variant seems to offer consistent results irrespective of allow-deny ratio. In the validation column, accuracy metrics reaches the lowest value of 86% (performance obtained for 10 Users “Allow” and 10 Users “Deny”), while the test column always surpasses it. It can be noticed that, performance on the test “deny” exclusive data can reach up to 93% accuracy. All categories were used to train the models in both scenarios. The subjects in the training group had an identical distribution of category instances, ensuring that each subject contained the same number of instances per category. These results are promising considering that current state of the art approaches tend to deal with simpler tasks. For example, in Bidgoly et al. (2022), their “allow” group consists of just one subject and impostors are always compared against that single person. This way they achieve around 98% accuracy.

### 3.4 User identification

For the last task we aimed to identify all 24 users. Similar to the previous scenario, all categories were used and we made sure that data from all participants is present in both train and test set. Data from each participant was split in 80% for training and the rest for testing. For this task we used our data to train EEGNet (with modifications as described in the first paragraph of Section 3) and to train a model as described in Maiorana (2020). As seen in Table 5, the personal EEG signature is consistently detected by EEGNet. From the pool of 24 people, the system can identify 11 with an accuracy of over 97% and 18 with an accuracy of at least 95%. The worst result, 87.08%, is obtained for U11 although the performance still maintains a high threshold. Therefore, for this task we obtained an overall mean accuracy of 96.28%. The second method, Maiorana (2020), yields relatively similar results, with a slightly lower mean accuracy of 94.78%.

**Table 5:** Accuracies [%] for EEG based user identification (24 Users).

**User**	**EEGNet**	**Maiorana (2020)**	**User**	**EEGNet**	**Maiorana (2020)**	**User**	**EEGNet**	**Maiorana (2020)**
U1	99.41 ± 7.84e-3	98.83 ± 0.66e-2	U9	97.84 ± 3.14e-2	94.58 ± 4.24e-2	U17	98.64 ± 1.56e-2	97.45 ± 1.42e-2
U2	100 ± 0	98.03 ± 1.24e-2	U10	94.96 ± 6.24e-2	97.62 ± 2.04e-2	U18	87.91 ± 6.21e-2	84.08 ± 6.43e-2
U3	96.07 ± 2.32e-2	99.81 ± 0.23e-2	U11	87.08 ± 2.20e-1	92.26 ± 7.66e-2	U19	98.14 ± 2.41e-2	91.59 ± 0.33e-1
U4	97.98 ± 2.45e-2	92.42 ± 5.32e-2	U12	96.68 ± 3.68e-2	91.29 ± 3.75e-2	U20	96.35 ± 5.65e-2	97.12 ± 1.97e-2
U5	99.71 ± 3.92e-3	97.35 ± 2.34e-2	U13	89.81 ± 6.68e-2	95.28 ± 2.45e-2	U21	94.51 ± 6.97e-2	94.56 ± 5.43e-2
U6	99.51 ± 7.59e-3	93.20 ± 2.88e-2	U14	95.00 ± 5.81e-2	96.04 ± 2.62e-2	U22	94.06 ± 7.53e-2	99.38 ± 0.60e-2
U7	98.53 ± 1.07e-2	95.33 ± 1.28e-2	U15	95.49 ± 5.28e-2	95.63 ± 2.75e-2	U23	99.90 ± 1.96e-3	94.83 ± 3.43e-2
U8	96.87 ± 3.26e-2	86.27 ± 4.29e-2	U16	99.50 ± 7.75e-3	94.07 ± 3.42e-2	U24	96.86 ± 4.65e-2	98.08 ± 1.37e-2

It is worth noting that achieving accuracies as high as 100% for some users is a notable achievement, reflecting the classifier's ability to perform exceptionally well when provided with clean, high-quality neural signals. For users with lower accuracies, factors such as residual noise from subtle movement artifacts and variations in electrode impedance may still affect the data, even after preprocessing. These results highlight the inherent challenges of EEG classification while demonstrating the strength of the system in handling high-quality data effectively.

Nonetheless, the impersonal categories, can result in EEG patterns that are similar up to a degree. For example, “hiking” category was liked by over two-thirds of participants. Thus, this category holds less value in discriminating between subjects (the model might be more inclined to learn characteristics of general liking, rather than specific EEG pattern that are participant specific). Additionally, due to inherent differences in EEG response, some users might exhibit more subtle variations when exposed to different stimuli. Therefore, users whose EEG activity is relatively constant might pose a higher challenge to the classifier.

This experiment shows the great potential of developing highly sophisticated human authentication systems based on the sole unique human marker: neural electrical activity. Also, the necessary stimulus to elicit such a signature is minimal and easy to replicate: visualizing an image on a screen. In addition, the reported performance is congruent with current state of the art results in EEG identification problems. For example, the worked described in (Mao et al., 2017) reports 97% accuracy, with the note that they used data from a driving fatigue experiment. Their acquisition paradigm implies that subjects were highly engaged, thus the elicited EEG response was more prominent. When (Mao et al., 2017) tried to identify subjects when no specific stimuli are present (using the same database), their accuracy dropped at 90%.

It is worth emphasizing that these results are obtained with data coming from many users. Namely, data comes from 24 different people. None of the subjects had any condition that would imply easily differentiable EEG patterns (e.g., epilepsy, encephalopathy, etc.). In addition, the extensive artifact removal assures that the classifier does not learn the overlapping noise that may present discriminant characteristics. These 2 points accentuate the network capabilities to reliably discern between different EEG signatures.

## 4 Discussion

The above presented experiments showcase the dataset versatility in being part in various types of BCI related applications. In addition, the obtained results serve as benchmark for future improvements and enhancements. Even though the obtained accuracies are comparable with state-of-the art ones, it should be noted that there are still problems with inter-user generalization. This is most prominent in hobby classification. For this task we created some macro-categories in order to augment training data and increase classification capabilities. We employed such an approach because user invariant traits were still extremely difficult to find. In future works we intent to overcome this current limitation.

During the experiment, the images and categories were shown in the same order for all participants. As the first step in detecting neural predilection to hobby-related stimuli, we opted for a fixed presentation order rather than randomizing categories or images. Given the complexity of disentangling brain responses across 32 categories (each containing 32 images) and our goal of evoking a deeper, more sustained emotional response, this approach aimed to minimize data variability and enhance reliability and comparability across participants by leveraging the temporal dynamics of ERP responses. Thus, order effects or anticipation effects are present in this approach and further, the brain may still be processing a strong emotional stimulus when the next category appears. These effects have been partially covered by the baseline correction for removing lingering activity and the cross-validation approach that help mitigate order effects to some extent and helping to prevent the model from picking up spurious correlations. However, this is not solved entirely, since the model may still capture neural responses like fatigue, anticipation, or habituation, significantly different at the beginning vs. the end of the session. The next step toward a biometric application would be to randomize trials and categories (maybe choose, e.g., 3–5 images from a category in a block, instead of a single one for a stronger effect of a continuous emotion), to change the sequence across sessions (e.g., Day 1 vs. Day 2) for a more robust authentication, to help ensure the model learns biometric features rather than order-specific effects.

Nonetheless, it is worth mentioning that these are preliminary results which we think are valuable in the current EEG research field. EEG data is notoriously hard to classify in inter-subject applications (usually, models that work on one dataset will not work on another) so new experiments help to better shape this ever-improving domain. Furthermore, our work is also offering free access to the newly acquired hobby EEG dataset. As new EEG datasets are highly difficult to acquire and often access is being restricted by a paywall, we consider that this addition holds considerable value in the current research space.

Our primary aim in this study was to showcase the versatility and potential of the newly acquired database. To achieve this, we demonstrated its utility across four distinct applications: preference degree classification, category classification, person authentication, and person identification. We acknowledge that a deeper analysis of task-specific features would provide valuable insights; however, such an in-depth exploration falls beyond the scope of this paper. Therefore, we plan to explore task-specific feature analyses in a future study.

Due to the nature of the experiment, which involved low engagement and a relatively long acquisition time, there was a risk that the EEG data could be affected by drowsiness (Gu et al., 2022; Han et al., 2019). To address this, we analyzed the power spectral density (PSD) in the delta and theta bands. Even though we observed some sporadic occurrences of fatigue with influences in the theta range of 4–5 Hz, they are not consistent throughout the entire acquisition period and across all epochs. The details of this analysis are provided in Supplementary material V. Consequently, we are confident that the presented results reflect higher-level cognitive processes rather than drowsiness. A detailed frequency analysis of the influences of excitement, fear, and stress will be presented in a follow-up paper.

Also, our dataset was recorded in one session per user. This could predispose the recordings to contain session specific cues, and to encourage the classifier to identify sessions rather than users (problematic especially for person identification). In order to mitigate such an effect we took regular breaks and also took breaks when the subject requested. Not only did we stop the stimuli, but we also allowed the subject to walk around and stretch, while ensuring minimal movement of the EEG cap. After each break, the electrode impedances were re-checked and adjusted with conductive gel where necessary. These breaks taken during intra-session recordings, even mild, can alter brain activity and physiological states. For instance, they can increase alertness and change the participant's mental state; which would be reflected in the EEG data after the break. Thus, even though the recordings were not conducted in technically separate sessions, the breaks could allow session–specific cues—such as mood influences—to change or dissipate. In addition, this current work is preliminary and we have planned to complement the study with additional sessions for higher reliability.

Moreover, our EEG dataset was acquired after extensive research on common hobby predilections. The categories where chosen after we compiled results from of a survey we conducted (details in Supplementary material I). This way, the shown stimuli are relevant and can be integrated in other applications. In addition, the high number of subjects is conducive for inter-subject EEG analysis paradigms.

## 5 Conclusion

EEG analysis is a dynamic field that holds tremendous promise for advancing both medical and artificial intelligence based applications which are aimed at evolving the overall understanding of the human psyche. This paper introduces a new EEG database containing neurological responses to popular hobbies, reference categories and images with significant personal importance. To the best of our knowledge, the paradigm of focusing on personal hobbies in order to tackle the possibility of extracting a digital biometric signature has never been explored before.

In this paper we offer 4 possible applications that can be developed starting from our proposed database. We report results for: emotion and category classification as well as binary authentication and user identification. Beside presented results, exhaustive testing is described in Appendix IV.

## Data Availability

The datasets presented in this study can be found in online repositories. The names of the repository/repositories and accession number(s) can be found in the article/Supplementary material.
