# Supplementary material for: Tackling the possibility of extracting a brain digital fingerprint based on personal hobbies predilection
Source: Front Neurosci. 2025 Mar 12;19:1487175. doi: 10.3389/fnins.2025.1487175 (PMC11937079; doi:10.3389/fnins.2025.1487175)
Supplement: Supplementary file 1 [file Data_Sheet_1.pdf]

Supplementary Material

I SURVEY

The 26 hobby categories, which were shown while collecting EEG data, were selected after a previous survey. The aim was to find the most common hobbies and interests among people.

There was a number of 96 respondents aged between 18 and 45, with a median value of about 30 years old. The hobbies we chose were the ones that emerged as the most popular during the survey (popularity of at least 8%). Besides that, we also added extreme sports (1.04%) to introduce variety.

The poll had 34 categories to choose from, including an ”other” category to fill in, if necessary. Examples from ”other” that appeared in the survey include: blogging/vlogging, studying languages, diorama modeling, illusionism, Greek mythology, etc.

The hobby categories, that were explicitly in the survey, but did not make it to the final study were: puzzle, origami, sewing, singing, architecture, equestrianism, pilotage and self-defense sports.

The occupation of the demographic was similar to the one present in the EEG data, meaning mostly IT (70%) complemented with people working in economy, law, photography, etc.

Survey results are briefly presented in the following (the percentage in brackets marks the proportion of people that chose that particular hobby/interest):

1. animals: pets, domestic, wild animals (25%)
2. team sports activities (basketball, football, tennis, ping-pong, etc.) (25%)
3. food/cooking (23.96%)
4. video games (20.83%)
5. board games (chess, backgammon, etc.) (19.79%)
6. DIY (Do-it-yourself)/construction/repairing (18.75%)
7. photography (18.75%)
8. water sports activities (swimming, surfing, kite-surfing, kayaking, maritime navigation) (16.67%)
9. gardening (14.58%)
10. cars/drifting/karting/F1 (14.58%)
11. hiking, climbing, rock climbing (50%)
12. yoga/meditation (12.5%)
13. musical instruments (11.46%)
14. art (painting, drawing) (11.46%)
15. fashion, clothes (11.46%)
16. body-building (9.38%)
17. dance (8.33%)
18. extreme sports (air sports) (1.04%)
19. travelling (53.13%)
20. reading (41.67%)

21. individual sport activities on wheels (road cycling, mountain biking, skateboarding, roller-skating, etc.) (42.71%)
22. movies (34.38%)
23. cardio, running, fitness (34.38%)
24. cultural activities: theater (acting), museums, opera, shows, stand-up comedy (29.17%)
25. technology (technological devices, not software) (31.25%)
26. winter sports activities (skiing, snowboarding, ice-skating, etc.) (27.08%)

II IMAGE AUTHORS

The authors for the pictures in Figure 2 (main text) are as follows: Animals: Adam Bignell and Wynand van Poortvliet, Team sports: Julian Schiemann and kevin turcios, Food: 8-Low Ural and Zac Cain, Video games: Ella Don and Jetshoots.com, Board games: Aedrian and Robbin Wong, DIY: Annie Spratt and subvertivo _lab, Photography: Artur Tumasjan and Erik Mclean, Water sports: Gentrit Sylejmani and Jeremy Bishop, Gardening: Benjamin Combs and GreenForce Staffing, Cars: Alexandre Bury and Campbell, Hiking: Toomas Tartes and Char Beck, Yoga: Avrielle Suleiman and Eneko Uruñuela, Musical instruments: Daniel Robert Dinu and Franki Chamaki, Art: Customerbox and Mick Haupt, Fashion: John Cameron and Michael Lee, Body building: Anastase Maragos and Benjamin Klaver, Dance: Ali Rizvi and Danielle Cerullo, Extreme sports: Kamil Pietrzak and Linus Mimietz, Travel: Ashim D'Silva and Mantas Hesthaven, Reading: Christian Wiediger and Chuttersnap, Sports with wheels: Isaiah Bekkers and Keagan Henman, Movies: Michael Marais and Jake Hills, Cardio: Cameron Venti and Nomadic Julien, Cultural: alevision.co and Alex Simpson, Technology: Lauren Mancke and Magnus Engø, Winter sports: Bradley King and Yann Allegre.

III HARDWARE AND SOFTWARE SETUP

The data was acquired with 33 gel electrodes, in monopolar montage, with mastoid references. The EEG sensors were distributed according to the extended 10–20 system of Neuroscan's 128-channels Quik-Cap (configuration illustrated in Figure S1). In terms of hardware and utilities, we used Compumedics Neuroscan EEG system with Neuvo amplifiers.

The stimuli markers were synchronized by StimTracker and the slideshow paradigm was created with STIM2. Online data visualization and saving was done with Curry7 software.

Participants sat on a comfortable chair in front of a L27T-1 LED Fujitsu screen with 1680×1050 resolution and 60 Hz refresh rate. The distance between the subject's eyes and the screen was between 80 cm and 100 cm.

IV EXTRA RESULTS

This appendix contains results which are mentioned in the main text. Table S1 presents results for emotion classification when data for each subject is trained separately. This experiment was done a preliminary test in order to validate the model learning capabilities. It can be noted that in this case results are almost perfect for all users.

When adding data from all users, results tend to decrease in performance. Tables S2, S3 and S4 show the in extenso version of Table S5. In these 3 additional tables accuracies are separated also according to their

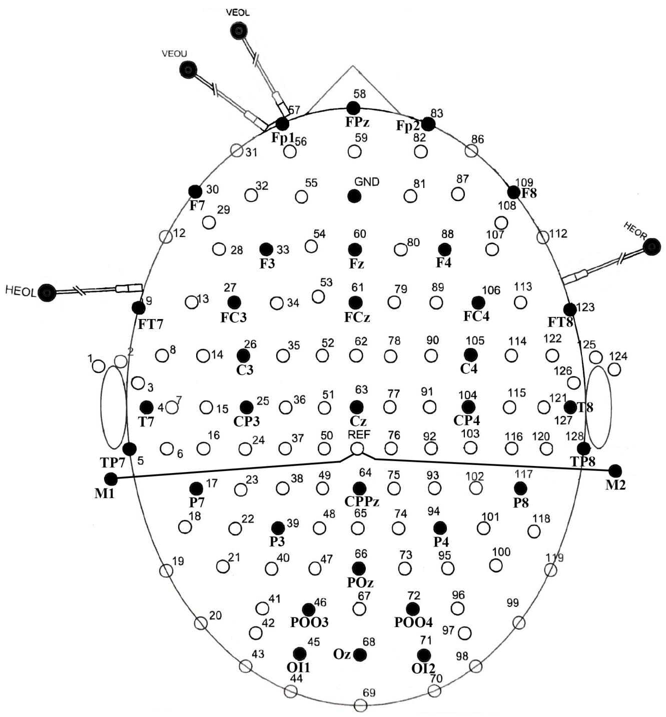

Figure S1: Electrodes configuration.

Table S1. EEGNet accuracy scores [%] for emotion classification (3 classes – like, neutral, dislike), one model for each user

2-folds	U1	U2	U3	U4	U5	U6	U7	U8
	99.51±9.77e-4	100±0	99.71±9.84e-4	99.70±1.01e-3	99.80±0	100±0	100±0	100±0
	U9	U10	U11	U12	U13	U14	U15	U16
	97.75±1.47e-2	100±0	100±0	100±0	100±0	100±0	100±0	99.19±0
	U17	U18	U19	U20	U21	U22	U23	U24
	100±0	100±0	100±0	100±0	100±0	100±0	100±0	100±0
3-folds	U1	U2	U3	U4	U5	U6	U7	U8
	100±0	100±0	99.90±1.39e-3	100±0	100±0	100±0	99.90±1.39e-3	99.90±1.39e-3
	U9	U10	U11	U12	U13	U14	U15	U16
	99.80±1.38e-3	100±0	100±0	100±0	100±0	100±0	100±0	99.90±1.42e-3
	U17	U18	U19	U20	U21	U22	U23	U24
	100±0	100±0	100±0	100±0	100±0	100±0	100±0	100±0
5-folds	U1	U2	U3	U4	U5	U6	U7	U8
	99.80±3.90e-3	100±0	100±0	100±0	100±0	100±0	100±0	100±0
	U9	U10	U11	U12	U13	U14	U15	U16
	100±0	100±0	100±0	100±0	100±0	100±0	100±0	100±0
	U17	U18	U19	U20	U21	U22	U23	U24
	100±0	100±0	100±0	100±0	100±0	100±0	100±0	100±0

respective label. For U5 there is no value for dislike accuracy because that user did not label any category as such.

Category classification proved to be a more challenging task. When trying to label 32 different classes the EEGNet proves to yield modest performances. On the hand SVM, barely manages to surpass the 3% random accuracy threshold. Therefore, we simplified the task by creating macro-categories. The macro-categories are comprised from categories that share a common theme. The main text contains results for 4 macro-categories classification. Tables S6 and S7 present results for 3 and 5 macro-categories. As expected, results vary depending on the number of classes considered.

Table S2. SVM results

	Dislike	Neutral	Like	Overall
U1	44.22+/-5.94	16.18+/-5.51	34.08+/-5.41	33.79+/-2.45
U2	29.55+/-5.85	19.73+/-5.29	66.41+/- 6.17	44.22+/-4.23
U3	12.13+/-3.72	39.97+/-6.88	62.81+/-5.78	51.52+/-2.71
U4	19.93+/-7.20	64.56+/-4.68	23.63+/-5.47	44.05+/-3.30
U5	N/A	41.11+/-4.37	49.12+/-5.02	44.82+/-3.51
U6	18.33+/-3.67	5.36+/-2.03	81.73+/-2.24	42.67+/-3.44
U7	27.49+/-5.95	30.43+/-3.24	56.38+/-2.77	46.33+/-2.08
U8	22.54+/-7.49	14.49+/-2.44	70.12+/-2.72	45.07+/-1.85
U9	34.55%+/-1.25	37.30+/-2.51	37.15+/-4.14	36.33+/-1.95
U10	26.38+/-2.53	19.83+/-3.82	65.57+/-4.99	37.60+/-1.95
U11	39.47+/-2.86	24.43+/-2.05	59.39+/-5.70	44.92+/-2.89
U12	20.54+/-5.70	33.50+/-5.34	41.35+/-5.10	32.65+/-4.76
U13	19.53+/-5.12	17.18+/-4.64	77.26+/-1.81	54.39+/-1.92
U14	16.11+/-3.34	22.68+/-7.37	76.74+/-2.07	46.67+/-4.54
U15	39.72+/-4.29	21.36+/-10.43	50.48+/-2.62	41.47+/-1.82
U16	23.78+/-2.58	55.79+/-5.48	13.93+/-2.96	30.24+/-3.20
U17	35.49+/-4.37	15.53+/-6.34	60.85+/-1.67	45.51+/-3.19
U18	32.33+/-1.96	26.07+/-7.47	41.78+/-6.32	32.75+/-3.19
U19	37.48+/-5.62	33.29+/-4.98	44.14+/-5.15	37.89+/-1.99
U20	20.66+/-9.61	30.09+/-6.31	54.56+/-4.33	39.69+/-1.45
U21	42.91+/-6.84	46.59+/-8.47	35.75+/-6.01	42.99+/-3.51
U22	40.80+/-6.00	14.55+/-4.40	66.32+/-4.48	39.71+/-3.41
U23	5.94+/-3.63	37.24+/-5.25	62.34+/-3.98	46.10+/-3.91
U24	49.95+/-2.18	31.53+/-6.20	31.26+/-5.13	40.72+/-1.91
Overall	33.48+/-1.25	32.22+/-0.70	55.81+/-0.99	41.74+/-0.25

Table S3. pyRiemann [44] results

	Dislike	Neutral	Like	Overall
U1	70.36+/-7.46	18.30+/-5.36	39.63+/-6.87	47.76+/-2.27
U2	47.77+/-5.28	43.13+/-10.11	69.06+/- 6.13	56.47+/-4.30
U3	36.61+/-15.21	45.90+/-3.85	71.24+/-8.38	60.36+/-7.52
U4	16.03+/-10.47	80.11+/-5.62	33.04+/-8.80	54.34+/-1.64
U5	N/A	54.37+/-6.59	56.09 +/-4.42	54.88+/-3.41
U6	23.20+/-10.95	39.97+/-11.80	78.56+/-6.45	51.37+/-2.60
U7	37.12+/-12.88	4.44+/-1.11	83.02+/-2.44	57.00+/-2.67
U8	23.98+/-11.18	52.13+/-9.77	78.22+/-9.41	62.76+/-4.45
U9	55.35+/-4.97	38.97+/-7.45	47.33+/-4.53	46.92+/-2.70
U10	70.33+/-6.50	40.49+/-9.10	58.17+/-5.87	55.86+/-4.14
U11	53.09+/-4.99	25.09+/-5.03	63.21+/-6.63	50.88+/-2.63
U12	46.53+/-5.58	29.67+/-7.31	64.40+/-6.70	47.51+/-3.36
U13	48.55+/-16.56	43.52+/-13.34	64.34+/-6.47	57.11+/-6.04
U14	4.41+/-5.59	64.86+/-4.89	87.59+/-2.28	65.31+/-4.28
U15	81.68+/-2.64	9.01+/-9.16	28.97+/-4.46	51.08+/-2.55
U16	18.33+/-5.50	45.86+/-3.08	48.37+/-3.75	38.81+/-1.78
U17	36.92+/-5.60	6.82+/-3.11	62.09+/-4.24	45.90+/-2.72
U18	78.53+/-4.88	44.92+/-5.20	29.60+/-9.00	55.81+/-3.51
U19	49.56+/-3.62	30.43+/-8.45	47.56+/-6.02	42.87+/-3.08
U20	26.63+/-6.56	24.32+/-7.53	75.77+/-4.99	48.85+/-2.27
U21	56.17+/-10.85	57.90+/-5.43	47.47+/-8.26	55.34+/-6.04
U22	64.13+/-11.20	23.62+/-7.85	30.30+/-11.15	43.36+/-6.16
U23	17.06+/-13.41	44.74+/-8.70	55.76+/-7.07	47.36+/-3.86
U24	52.64+/-15.72	48.45+/-9.26	36.13+/-5.81	52.35+/-3.42
Overall	51.50+/-1.63	42.38+/-1.23	60.35+/-1.42	52.07+/-0.68

V DROWSINESS ANALYSIS

Drowsiness onset can be noticed in delta (0.5–4 Hz) and theta (4–7 Hz) bands. As we already removed frequencies below 3 Hz, we continued to monitor progression of PSD in the 3–7 Hz band. Even though we observed some sporadic occurrences of fatigue with influences in the theta range of 4–5 Hz, they are not consistent throughout the entire acquisition period and across all epochs. As illustrated in Fig. S4, the power in 3–7 Hz does not suffer significant modification during the acquisition, considering the mean over all participants. Also, to showcase that the results presented in Fig. S4 are consistent across participants, we present in Fig. S7 the PSD from the same epochs for an individual participant.

Table S4. EEGNet results

	Dislike	Neutral	Like	Overall
U1	69.67+/-9.31	69.69+/-7.16	71.66+/-4.85	70.61+/-3.66
U2	75.03+/-9.65	77.33+/-12.19	86.02+/- 4.68	80.88+/-2.32
U3	79.83+/-18.92	79.80+/-4.21	82.84+/-2.01	81.36+/-2.34
U4	60.15+/-10.89	83.76+/-4.95	73.43+/-12.25	76.50+/-3.86
U5	N/A	81.71+/-4.80	84.17+/-4.25	83.01+/-2.78
U6	84.11+/-9.24	88.02+/-12.09	90.37+/-12.05	87.27+/-3.61
U7	84.82+/-10.00	87.42+/-5.13	90.93+/-3.87	89.33+/-2.10
U8	84.18+/-7.84	90.32+/-1.25	85.94+/-4.47	87.38+/-1.27
U9	64.11+/-11.05	61.00+/-11.37	67.08+/-14.22	64.44+/-6.97
U10	89.49+/-8.57	93.34+/-5.82	91.78+/-8.97	91.40+/-3.64
U11	89.46+/-3.46	81.51+/-9.87	85.37+/-5.24	85.46+/-3.84
U12	79.02+/-10.10	87.70+/-7.18	81.97+/-8.05	83.48+/-2.52
U13	90.34+/-5.47	89.77+/-7.67	93.87+/-2.88	92.33+/-3.15
U14	74.83+/-14.07	91.76+/-6.71	92.18+/-7.08	89.37+/-2.88
U15	80.05+/-9.30	71.15+/-15.07	85.84+/-6.13	81.86+/-3.21
U16	71.58+/-6.63	69.88+/-6.82	69.64+/-3.86	70.47+/-2.65
U17	80.06+/-8.40	77.15+/-3.73	87.77+/-7.51	83.89+/-5.03
U18	82.21+/-5.36	91.92+/-5.71	80.16+/-4.01	84.46+/-3.19
U19	79.42+/-10.65	71.55+/-3.12	91.75+/-9.83	80.58+/-6.89
U20	56.95+/-12.4	62.31+/-6.45	77.33+/-3.86	67.92+/-1.79
U21	84.27+/-4.31	90.03+/-5.18	83.68+/-8.90	86.29+/-2.50
U22	94.19+/-2.22	91.98+/-8.55	88.14+/-14.55	91.93+/-3.12
U23	59.75+/-18.64	82.36+/-10.72	83.30+/-7.90	80.47+/-4.64
U24	86.84+/-5.4	92.58+/-4.57	78.54+/-13.28	87.41+/-3.68
Overall	80.07+/-3.74	82.44+/-2.85	84.22+/-3.76	82.44+/-1.27

Table S5. Emotion classification; 3 classes: *dislike*, *neutral* and *like*

	SVM	pyRiemann [44]	EEGNet
U1	33.79+/-2.45	47.76+/-2.27	70.61+/-3.66
U2	44.22+/-4.23	56.47+/-4.30	80.88+/-2.32
U3	51.52+/-2.71	60.36+/-7.52	81.36+/-2.34
U4	44.05+/-3.30	54.34+/-1.64	76.50+/-3.86
U5	44.82+/-3.51	54.88+/-3.41	83.01+/-2.78
U6	42.67+/-3.44	51.37+/-2.60	87.27+/-3.61
U7	46.33+/-2.08	57.00+/-2.67	89.33+/-2.10
U8	45.07+/-1.85	62.76+/-4.45	87.38+/-1.27
U9	36.33+/-1.95	46.92+/-2.70	64.44+/-6.97
U10	37.60+/-1.95	55.86+/-4.14	91.40+/-3.64
U11	44.92+/-2.89	50.88+/-2.63	85.46+/-3.84
U12	32.65+/-4.76	47.51+/-3.36	83.48+/-2.52
U13	54.39+/-1.92	57.11+/-6.04	92.33+/-3.15
U14	46.67+/-4.54	65.31+/-4.28	89.37+/-2.88
U15	41.47+/-1.82	51.08+/-2.55	81.86+/-3.21
U16	30.24+/-3.20	38.81+/-1.78	70.47+/-2.65
U17	45.51+/-3.19	45.90+/-2.72	83.89+/-5.03
U18	32.75+/-3.19	55.81+/-3.51	84.46+/-3.19
U19	37.89+/-1.99	42.87+/-3.08	80.58+/-6.89
U20	39.69+/-1.45	48.85+/-2.27	67.92+/-1.79
U21	42.99+/-3.51	55.34+/-6.04	86.29+/-2.50
U22	39.71+/-3.41	43.36+/-6.16	91.93+/-3.12
U23	46.10+/-3.91	47.36+/-3.86	80.47+/-4.64
U24	40.72+/-1.91	52.35+/-3.42	87.41+/-3.68
Overall	41.74+/-0.25	52.07+/-0.68	82.44+/-1.27

Table S6. Results for 3 macro-category classification

Aggregate category	Composing categories	Accuracy	Overall performance
sports	team sports<br>water sports<br>hiking<br>body building	88.04±2.03%	87.39±1.32%
reference	traditional patterns<br>fractals<br>Brownian fractals<br>mono-color	88.05±2.14%	
serenity/calm	animals<br>personal images<br>musical instruments<br>DIY	86.16±1.74%	

Table S7. Results for 5 macro-category classification

Aggregate category	Composing categories	Accuracy	Overall performance
physical movement	water sports<br>hiking<br>body building	61.80±5.91%	70.15±1.58%
reference	fractals<br>Brownian fractals<br>mono-color	77.46±2.84%	
serenity/calm	animals<br>personal images<br>musical instruments	76.42±1.10%	
games	video games<br>team sports<br>board games	73.80±3.13%	
modern electronics	technology<br>photography<br>cars	61.76±1.65%	

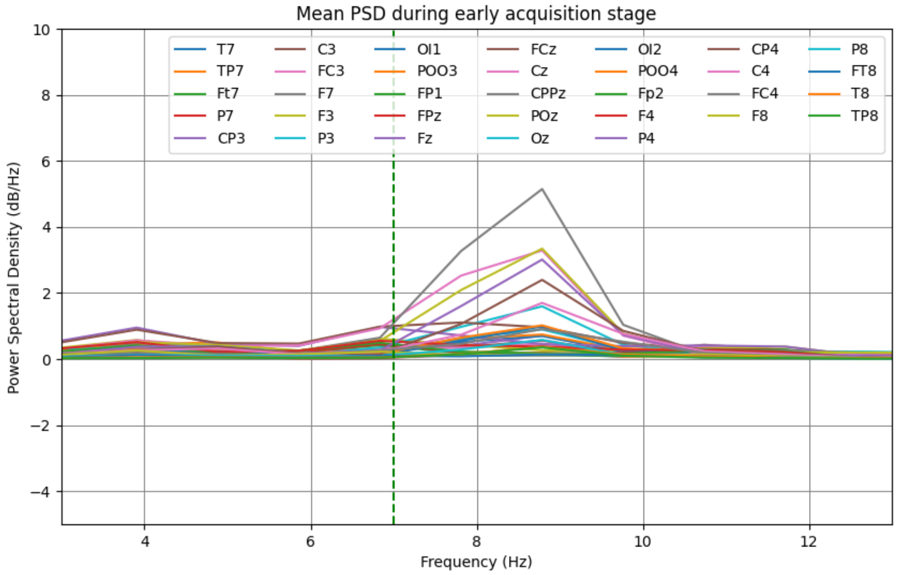

Figure S2: Epoch from the first category shown

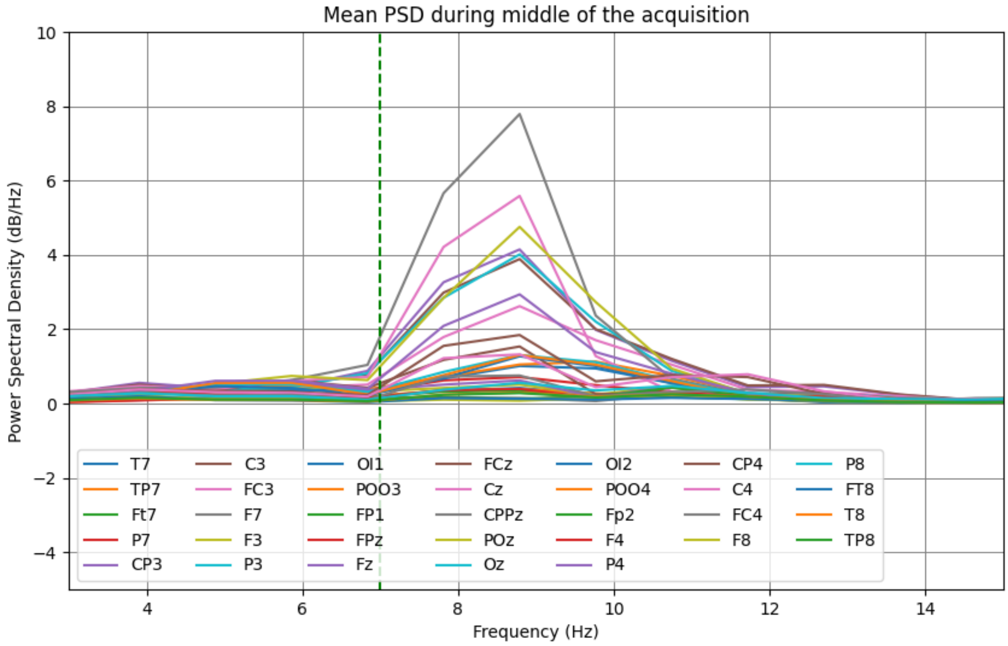

Figure S3: Epoch from the 16th category shown

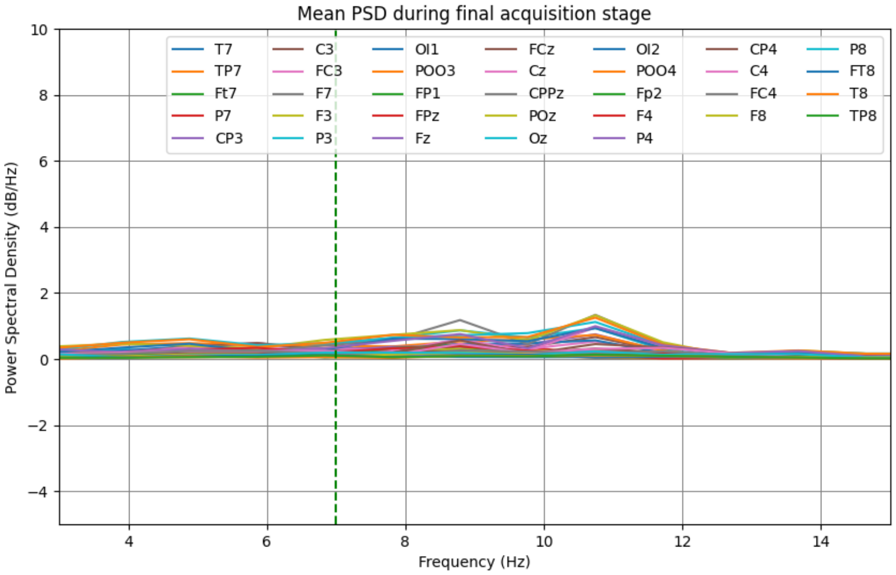

Figure S3: Epoch from the last category shown

Figure S4: Mean PSD evolution during the acquisition. Green dotted line represents the boundary between theta and alpha band. PSD was computed as the mean between subjects whose acquisitions were complete (i.e., no epochs were eliminated during preprocessing).

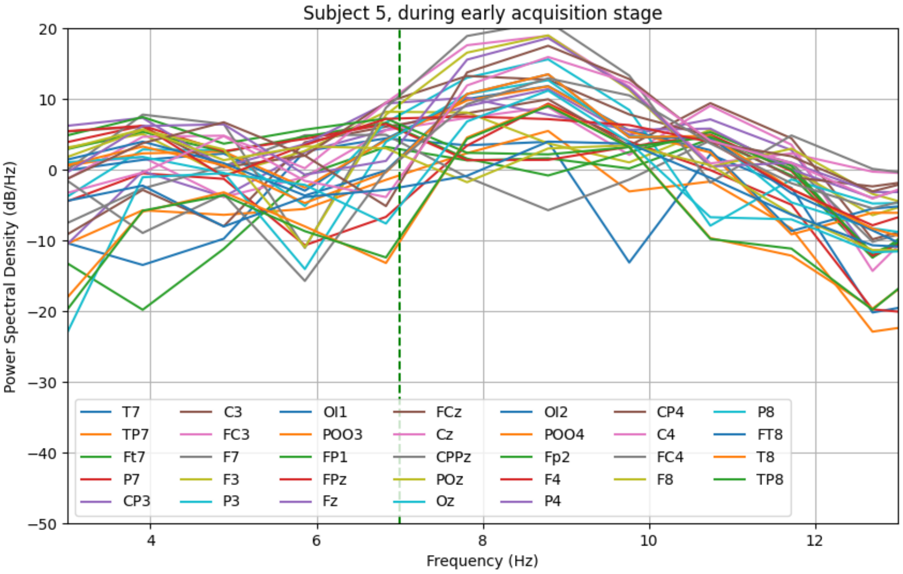

Figure S5: Epoch from the first category shown

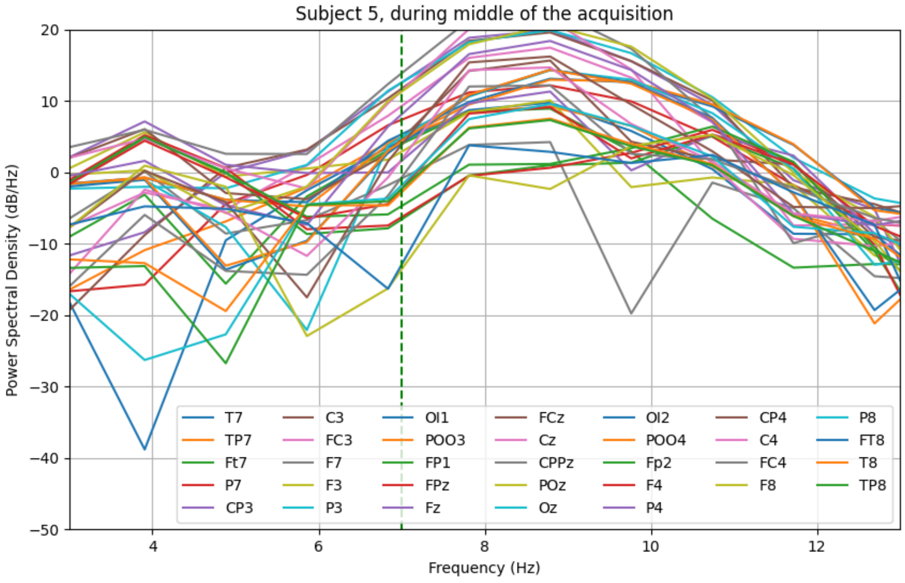

Figure S5: Epoch from the 16th category shown

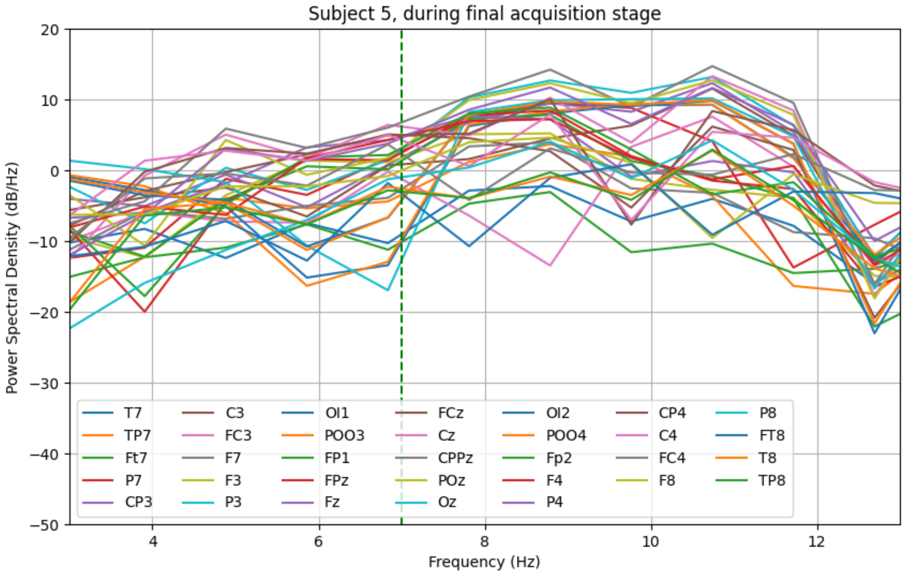

Figure S6: Epoch from the last category shown

Figure S7: PSD computed for subject 5. Data shown is from the same epochs as Fig. S4
